# Effect of temporary cements and their removal methods on the bond strength of indirect restoration: a systematic review and meta-analysis

**DOI:** 10.1007/s00784-022-04790-6

**Published:** 2022-11-24

**Authors:** Jingyu Ding, Yifu Jin, Shanshan Feng, Huan Chen, Yanyan Hou, Song Zhu

**Affiliations:** Department of Prosthodontics, Hospital of Stomatology, Jilin University, 1500 Qinghua Road, Chaoyang District, Changchun, 130012 Jilin China

**Keywords:** Immediate dentin sealing, Bond strength test, Dental bonding, Indirect restoration, Dental cements, Temporary dental restoration

## Abstract

**Objectives:**

For a conventional indirect restoration, temporary cementation inevitably contaminated collapsed dentin collagen. The purpose of this review was to evaluate the optimal strategy for minimizing its negative effects.

**Material and methods:**

Databases such as PubMed, Web of Science, EMBASE, and the Cochrane Library were searched for in vitro studies, involving the influence of immediate dentin sealing (IDS), different temporary cements, and their removal strategies on dentin bond strength. The meta-analysis used the inverse variance method with effect method of the standardized mean difference and statistical significance at *p* ≤ 0.05. The *I*^2^ value and the *Q*-test were used to assess the heterogeneity.

**Results:**

A total of 14 in vitro trials were subjected to the meta-analysis. Within the study’s limitations, we assumed that IDS eliminated the negative effects of temporary bonding, achieving the comparable immediate bond strength with the control (*p* = 0.46). In contrast, under delayed dentin sealing (DDS), temporary cementation statistically decreased bond strength (*p* = 0.002). Compared with resin-based and non-eugenol zinc oxide cements, polycarboxylate and calcium hydroxide cements performed better on bond strength with no statistical difference from the control group (*p* > 0.05). Among the removal methods of temporary cements, the Al_2_O_3_ abrasion restored the decreased bond strength (*p* = 0.07) and performed better than hand instruments alone (*p* = 0.04), while pumice removal slightly reduced the bond strength in contrast with the control group (*p* = 0.05, 95% CI =  − 1.62 to 0).

**Conclusions:**

The choices of IDS, polycarboxylate and calcium hydroxide temporary cements, Al_2_O_3_ abrasion removal method were feasible and efficient to enhance the bond strength.

**Clinical relevance:**

It is worthwhile applying IDS technique, polycarboxylate and calcium hydroxide temporary cements during indirect restoration. The Al_2_O_3_ abrasion of cleaning dentin can minimize the negative effects of temporary cement.

**Supplementary Information:**

The online version contains supplementary material available at 10.1007/s00784-022-04790-6.

## Introduction

With the advances in adhesive technology and prosthetic material, the demand for indirect restoration is increasing with the advantages of superior aesthetic and mechanical properties over direct restoration [1]. However, during the first visit, indirect restoration involves multiple procedural steps including tooth preparation, impression making, and temporary restoration [2, 3]. After an inevitable delay of fabricating laboratory restoration, at the second visit, the temporary restoration and cement are removed, and the final restoration is luted by a luting system [4]. At the moment, this conventional technique of dentin bonding prior to final restoration was referred to delayed dentin sealing (DDS) [5], which could lead to bacterial leakage and dentin hypersensitivity due to unsealed dentin during the temporary period [6, 7]. Aside from the impact of the temporary period on interface quality [5], the collapsed dentin collagen fibers contaminated by blood and temporary cement would cause the difficulty of subsequent adhesive penetrating and hybrid layer forming, bringing about inferior bond strength compared with freshly cut dentin [4, 8].

Based on clinical restrictions mentioned above, immediate dentin sealing (IDS) has emerged to seal the freshly cut dentin immediately after tooth preparation, when non-collapsed dentin collagen fibers would let the adhesive penetrate easier and prepolymerize without the pollution [8]. Meta-analyses have shown that the IDS technique could enhance the bond strength of resin-based restoration regardless of the adhesive strategy used [4], but lacking clinical trials to prove its advantage of reducing postoperative sensitivity [9]. Therefore, IDS is promising to mitigate the negative effects of temporary cement and temporary period on bond strength compared with DDS, which has not been systematically analyzed yet.

In addition, various strategies for minimizing the negative effects of temporary cement have been proposed, including effective removal ways and optimal selection of temporary cements, which have been shown to affect bond strength substantially [2, 5]. The contamination of blood or saliva could be resolved by primer re-application or water rinsing [10], but additional removal ways were required to clean temporary cement. It has been suggested that adequately removing would not affect immediate bond strength but undermine the bond durability [11, 12]. Therefore, taking appropriate clinical measures was imperative but controversial [2]. In terms of mechanical cleaning ways alone, Santos found that Al_2_O_3_ abrasion resulted in notably higher bond strength than pumice slurry [13], while Özcan revealed that there was no significant difference between them [14]. Similarly, considering various temporary cements, resin-based cement was discouraged due to its removal challenge and bond strength decline [5, 15], whereas other scholars came to the opposite conclusion [16]. But it was widely acknowledged that zinc oxide cement with eugenol inhibited polymerization, regardless of the adhesive system and bond strength test modality [15, 17].

As a result, aiming at drawing the suitable strategies for minimizing the negative effects of temporary cementation, the current study would conduct a systematic review of the role of IDS and the influence of various temporary cements and their removal methods on the bond strength. The null hypothesis stated neither the adoptions of IDS nor various temporary cements and their cleaning ways had difference in bond strength after temporary restoration.

## Material and methods

This systematic review was conducted according to the PRISMA statement [18]. The protocol was registered in the PROSPERO international database (CRD42022325984). PICOS elements for a systematic review were as follows: participant (P): dentin of healthy human permanent teeth for indirect restoration; intervention (I): temporary cementation with temporary cement removal, applying the IDS or DDS techniques; comparison (C): comparative studies with at least one control group without temporary cementation (blank control) or another method of temporary cement removal (positive control); outcome (O): the bond strength, including microtensile, microshear, or shear bond strength (MTBS, MSBS, and SBS); study types (S): in vitro and in situ laboratory studies.

The literature search was done by 2 independent reviewers until April 8, 2022, in 4 different databases: MEDLINE (PubMed), Web of Science, EMBASE, and the Cochrane Library, with no restriction for language and publication dates. Grey literature was searched in the grey source index of greynet. Search terms were constrained in title/abstract, except for Mesh terms. The search strategy in PubMed is shown in Table 1. Other databases’ search strategies are attached in supplementary material.

**Table 1:** Search strategy used in PubMed (MEDLINE)

Search terms
#1: Bonding OR bond OR bonding efficacy OR dental bonding OR bond strength OR bonding effectiveness OR bonding performance OR bond performance OR adhesive properties OR micro-tensile strength OR microtensile strength OR microtensile bond strength OR bonding properties OR microshear bond strength OR shear bond strength
#2: Dentin* OR dentin [MESH]
#3: Provisional cement* OR temporary cement* OR interim cement* OR temporary restoration* OR provisional restoration* OR interim restoration*
#4: #1 and #2 and #3

### Inclusion and exclusion criteria

For qualitative synthesis, we only included in vitro laboratory studies that evaluated the effects of temporary cementation or different temporary cement removal strategies on bond strength. Studies containing the following criteria were excluded in this review: (1) participants were non-human animal dentin, such as bovine dentin; (2) studies where zinc oxide and eugenol were used as temporary cement; (3) researches without a temporary period failed to realistically simulate the clinical process, so they were excluded; (4) small sample size: tooth number was less than 3 or sticks for MTBS were less than 24 per group [19]; (5) research subjects were various temporary sealing materials used in endodontics, such as glass ionomer.

### Risk of bias

After searching in the database, we exported the articles to remove duplicate articles. Based on the titles and abstracts, we carried out an initial screening of the retrieved studies. We reassessed the remaining full texts and only included those that met inclusion criteria. To assess the reliability of the findings, we used the parameters shown in Table 2. If the authors mentioned the parameter, the study received a “YES” for that specific parameter. In contrast, it gained a “NO.” The risk of bias was classified based on the sum of “YES” responses: 1 to 3 indicated a high risk, 4 to 6 indicated a medium risk, and 7 to 9 indicated a low risk [4].

**Table 2:** Bias risk assessment

Study	Specimen randomization	Single operator	Operator blinded	Standardized specimens	Failure mode	Manufacturer’s instructions	Sample size calculation	Caries free	Control group	Risk of bias
Maciel et al. 2021	NO	NO	NO	YES	YES	YES	NO	YES	YES	Medium
Hayashi et al. 2019	NO	NO	NO	YES	YES	YES	NO	YES	YES	Medium
Augusti et al. 2017	YES	YES	NO	YES	YES	YES	NO	YES	YES	Low
Özcan et al. 2015	YES	YES	NO	YES	YES	YES	YES	NO	YES	Low
Tajiri-Yamada et al. 2020	YES	NO	NO	YES	YES	NO	NO	NO	YES	Medium
Hironaka et al. 2018	NO	NO	NO	YES	YES	YES	NO	YES	YES	Medium
Abo-Hamar et al. 2005	YES	YES	NO	YES	YES	YES	NO	YES	YES	Low
Altintas et al. 2011	YES	YES	NO	YES	YES	YES	NO	YES	YES	Low
Lima et al. 2017	YES	NO	NO	NO	YES	YES	NO	YES	YES	Medium
Dillenburg et al. 2009	YES	NO	NO	YES	YES	YES	NO	NO	YES	Medium
Erkut et al. 2007	NO	NO	NO	NO	NO	YES	NO	YES	YES	High
Yap et al. 2001	YES	NO	NO	NO	YES	YES	NO	YES	YES	Medium
Fiori-Júnior et al. 2010	YES	YES	NO	YES	NO	YES	NO	YES	YES	Medium
Chiluka et al. 2017	YES	NO	NO	NO	NO	YES	YES	NO	YES	Medium
Carvalho et al. 2014	YES	NO	NO	YES	YES	YES	NO	YES	YES	Medium
Bagis et al. 2011	YES	NO	NO	YES	YES	YES	NO	YES	YES	Medium
Santos et al. 2011	YES	NO	NO	YES	NO	YES	NO	YES	YES	Medium
Januario et al. 2019	YES	YES	NO	YES	YES	YES	NO	YES	YES	Low
Breemer et al. 2019	YES	NO	NO	NO	YES	NO	NO	YES	YES	Medium
Falkensammer et al. 2014	NO	NO	NO	YES	YES	YES	NO	YES	YES	Medium
Zortuk et al. 2012	YES	NO	NO	YES	YES	YES	NO	YES	YES	Medium
Latta et al. 2005	No	No	No	No	No	No	No	No	Yes	High

### Statistical analysis

Relevant data from the studies were extracted using Microsoft Word 2010 sheets. To retrieve the absent information, we contacted the authors of the included studies by e-mail. If they did not respond, we excluded the information [20]. Review Manager 5.4.1 (RevMan) was used to calculate the continuous data with the inverse variance method and effect method of the standardized mean difference. Statistical significance was measured using the *Z*-test (*p* ≤ 0.05). The statistical heterogeneity was assessed by the Cochran *Q*-test with *I*^2^ ≥ 50% considered as a suggestion of low-to-moderate heterogeneity transition. When *I*^2^ ≥ 50% existed among groups, the random-effects model was used; otherwise, we chose the fixed-effects model.

## Results

**Fig. 1:**
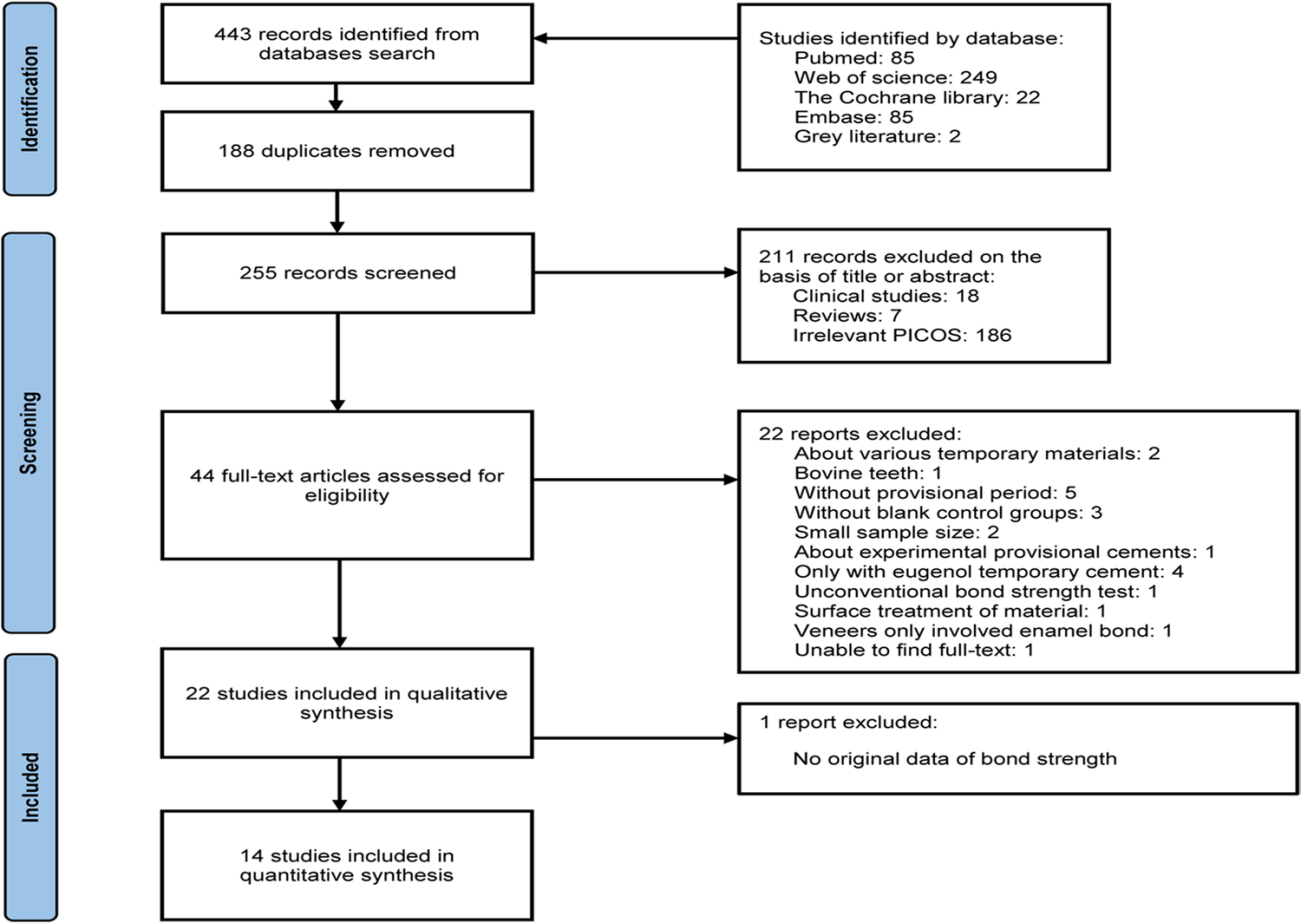
PRISMA flowchart of study selection

We found a total of 443 articles, where we screened 255, removing 188 duplicates. After we read the titles and abstracts, leaving 44 studies assessed for full text, we systematically reviewed 22 articles meeting the criteria and excluded 1 article because we failed to have access to the full text (Fig. 1). The risk of bias is shown in Table 2. All articles used English and human molars as samples. The comparisons with blank controls are shown in Table 3, and other comparisons among removal ways without blank controls are displayed in Table 4.

**Table 3:** Characteristics of the studies compared with blank controls

Author Sample size	Methodology	IDS or DDS?	Dentin bonding adhesive	Temporary cement	Temporary period time	Temporary cement removal method	Aging process
Maciel [21] *N* = 9 (teeth) 2021	MTBS	IDS	Etch-and-rinse system (Adper Scotchbond Multipurpose)	Non-eugenol zinc-oxide resin cement (RelyX Temp NE, 3 M)	1 week	Periodontal curette	Water for 48 h
						100 μm NaHCO_3_ sandblasting (5.51 bar; 10 s; 2.0 cm)	
						30 μm Al_2_O_3_ sandblasting (5.51 bar; 10 s; 2.0 cm)	
						Diamond bur	
				None	None	Blank control	
Hayashi [11] *N* = 15 (teeth) 2019	MTBS	IDS	All-in-one (Clearfil Universal Bond Quick) + flowable composite (Clearfil Majesty ES Flow)	Non-eugenol zinc-oxide cement (TempBond NE, Kerr)	1 week	Polishing brush	Cyclic load of 118 N over 90 cycles/min for 3 × 10^5^ cycles
				None	None	Blank control	
		DDS	None	TempBond NE, Kerr	1 week	Polishing brush	
				None	None	Blank control	
Fiori-Júnior [22] *N* = 10 (teeth) 2010	SBS	DDS	None	Zinc oxide-based cement (eugenol-free)	24 h	Hand excavator	Water for 24 h
				Calcium hydroxide-based			
				None	None	Blank control	
Augusti [23] *N* = 3 (teeth) *N* = 10 (specimen) 2017	MSBS	IDS	Etch-and-rinse adhesive (OptiBond FL)	Non-eugenol zinc-oxide cement (TempBond NE, Kerr)	24 h	Hand scaler	Water for 24 h
						50 μm Al_2_O_3_ air abrasion (20 s; 10 mm; 2.8 bar)	
						25 μm glycine air abrasion (20 s; 10 mm; 2.8 bar)	
						Liquid chemical solvent of D-limonene	
				Resin-based provisional agent (TempBond Clear, Kerr)	As above	Hand scaler	
						50 μm Al_2_O_3_ air abrasion	
						25 μm glycine air abrasion	
				None	None	Blank control	
Özcan [14] *N* = 3 (teeth) *N* = 12 (specimen) 2015	MSBS	IDS	Etch-and-rinse adhesive (OptiBond FL)	Eugenol-free zinc oxide cement (Freegenol, GC)	24 h	50 μm Al_2_O_3_ (2 bar;5 s; 10 mm)	Water for 24 h
						50 μm Al_2_O_3_ (3.5 bar; 5 s; 10 mm)	
						30 μm SiO_2_ (2 bar; 5 s; 10 mm)	
						30 μm SiO_2_ (3.5 bar; 5 s; 10 mm)	
						Prophylaxis paste using brush (1500 rpm; 15 s)	
						Pumice-water slurry using brush (1500 rpm; 15 s)	
				None	None	Blank control	
Abo-Hamar [20] *N* = 10 (specimen) 2005	SBS	DDS	None	Non-eugenol zinc-oxide cement (TempBond NE, Kerr)	1 week	30 μm alumina air-abrasion (4 bar; at 3 mm)	Water for 24 h
						Excavator	
				None	None	Blank control	
TAJIRI [24] *N* = 6 (teeth) *N* = 30 (specimen) 2020	MTBS	DDS	None	Polycarboxylate cement (HY-BOND)	1 week	Air scaler (20 s)	Water for 24 h, 1 month, or 6 months
						Rotating brush with water (20 s)	
						Air scaler (20 s) + phosphoric acid (20 s) + water rinsing (20 s) + NaClO (60 s)	
						Air scaler (20 s) + cleaner with MDP (10 s) + agitated with a micro-brush (10 s)	
				None	None	Blank control	
Altintas [3] *N* = 5 (teeth) *N* = 10 (specimen) 2011	SBS	DDS	None	Eugenol-free resin cement (Cavex)	1 week	Dental explorer	Water for 24 h
						Cleaning bur for 1 min	
				Calcium hydroxide cement (Dycal)	As above	Dental explorer	
						Cleaning bur for 1 min	
				Resin cement (TempBond Clear, GC)	As above	Dental explorer	
						Cleaning bur for 1 min	
				None	None	Blank control	
Erkut [25] *N* = 10 (teeth) 2007	SBS	IDS	Bonding agent (Single Bond)	Non-eugenol zinc-oxide resin cement (RelyX Temp NE, 3 M)	1 week	Scaler + fluoride-free pumice with water	Thermocycling × 1000 cycles between 5 °C and 55 °C + water for 1 week
			Resin-based (RelyX ARC one step)				
		DDS	None	As above	As above	As above	
				None	None	Blank control	
Hironaka [12] *N* = 10 (teeth) 2018	MTBS	IDS	Self-etching (Clearfil SE Bond 2) + layer of Protect Liner F	Non-eugenol zinc-oxide cement (TempBond NE, Kerr)	2 weeks	Explorer + pumice with water	Artificial saliva for 24 h
		DDS	None	As above	As above	As above	
				None	None	Blank control	
Dillenburg [26] *N* = 3 (teeth) 2009	MTBS	IDS	Etch and rinse (Adper Single Bond 2)	Non-eugenol zinc-oxide resin cement (RelyX Temp NE, 3 M)	2 d	AO: excavator + 50 μm Al_2_O_3_ (5.51 bar; 10 s; 2 cm) + air/water (10 s)	Artificial saliva for 24 h
					4 months		
					2 d	PA: excavator + 37% phosphoric acid (15 s) + air/water (15 s)	
					4 months		
					2 d	AO + PA: excavator + 50 μm Al_2_O_3_ (5.51 bar; 10 s; 2 cm) + PA	
					4 months		
				None	None	Blank control	
			Etch and rinse (Prime & Bond NT)	Non-eugenol zinc-oxide resin cement (RelyX Temp NE, 3 M)	2 d	AO	
					4 months		
					2 d	PA	
					4 months		
					2 d	AO + PA	
					4 months		
				None	None	Blank control	
Yap [27] *N* = 8 (teeth) 2001	SBS	DDS	None	Polycarboxylate eugenol-free cement	1 week	An ultrasonic scaler + pumice-water slurry	Water for 24 h
				None	None	Blank control	
Chiluka [28] *N* = 20 (teeth) 2017	SBS	DDS	None	Zinc oxide cement (eugenol-free)	1 week	Hand scaler	100% humidity for 24 h
					2 weeks		
				None	None	Blank control	
Carvalho [29] *N* = 5 (teeth) 2014	MTBS	DDS	None	Non-eugenol resin cement (RelyX Temp NE, 3 M)	1 week	Stainless steel spatula + pumice-water (60 s) + water stream (20 s)	Water for 24 h
				None	None	Blank control	
Bagis [30] *N* = 5 (teeth) 2011	MTBS	DDS	None	Zinc oxide cement (TempBond NE, Kerr)	1 week	Excavator	Water for 24 h
				None	None	Blank control	
LIMA [16] *N* = 6 (teeth) *N* = 12 (specimen) 2017	MSBS	IDS	Etch and rinse (Adper Scotchbond Multipurpose)	Resin cement (RelyX Temp NE, 3 M)	7 d	Hand scaler	100% humidity environment for 24 h
				Resin cement (Provitemp)			
				None	None	Blank control	
Latta [31] *N* = 10 (teeth) 2005	MSBS	IDS	Etch and rinse (Prime & Bond NT)	Non-eugenol zinc oxide cement (Nogenol, GC)	7 d	Dental instrument	Water for 24 h
						Dental instrument + phosphoric acid (PA)	
			Self-etching (Clearfil SE Bond)	As above	As above	Dental instrument	
						Dental instrument + PA	
		DDS	None	As above	As above	Dental instrument	
				None	None	Blank control

**Table 4:** Characteristics of the studies compared with positive controls

Author Sample size	Methodology	IDS or DDS?	Dentin bonding adhesive	Temporary cement	Temporary period time	Temporary cement removal method	Aging process
Santos^13^ *N* = 13 (specimen) 2011	SBS	DDS	None	Non-eugenol zinc-oxide cement (TempBond NE, Kerr)	1 week	Excavator (10 s)	Water for 24 h
						0.12% chlorhexidine digluconate (10 s)	
						40% polyacrylic acid (10 s)	
						Pumice slurry (10 s)	
						50 μm Al_2_O_3_ particles (6 bar; 10 s; 2 cm)	
Januario^29^ *N* = 8 (teeth) 2019	SBS	DDS	None	Non-eugenol zinc-oxide resin cement (RelyX Temp NE, 3 M)	15 d	Excavator + air–water rinse (10 s; 5 mm)	Water for 90 d
						Excavator + pumice paste with brush (10 s)	
						Excavator + 50 μm Al_2_O_3_ air abrasion (2.5 bar; 20 s; at 90°; 10 mm)	
						Excavator + NaHCO_3_ air abrasion (2.5 bar; 20 s; at 90°; 10 mm)	
						Excavator + glycine powder air abrasion (2.5 bar; 20 s; at 90°; 10 mm)	
Breemer^31^ *N* = 10 (teeth) 2019	SBS	IDS	Self-etching (Clearfil SE Bond) with 3 strategies	Non-eugenol zinc-oxide cement (TempBond NE, Kerr)	2 weeks	Pumice slurry (10 s)	Thermocycling × 10,000 cycles, between 5 °C and 55 °C
						Pumice + 30 μm silicoated Al_2_O_3_ (10 mm, angle 45 degrees, 2 bar) + silane coupling agent	
			Etch-and-rinse (OptiBond FL) with 3 strategies	As above	As above	Pumice slurry (10 s)	
						Pumice + 30 μm silicoated Al_2_O_3_ + silane coupling agent	
		DDS	None	As above	As above	Pumice slurry (10 s)	
Falkensammer^32^ *N* = 11 (teeth) 2014	SBS	IDS	Self-etching adhesive (AdheSE)	Non-eugenol zinc-oxide cement (TempBond NE, Kerr)	1 week	Fluoride-free pumice paste (1000 rpm, 5 s)	Saline solution for 24 h
						Silicoated Al_2_O_3_ air abrasion (5 s; 2 cm; 90 degrees) (dry condition)	
						Glycine air abrasion (5 s; 2 cm; 90 degrees) (wet condition)	
						CaCO_3_ air abrasion (5 s; 2 cm; 90 degrees) (wet condition)	
		DDS	None	As above	As above	Blank control	
Zortuk^33^ *N* = 9 (teeth) 2012	SBS	DDS	None	Eugenol-free resin cement (Cavex)	2 d	Dental explorer	Thermocycling × 5000 cycles; between 5 °C and 55 °C
						Pumice under water (1 min; 5000 rpm)	
						Bur (1 min; 5000 rpm)	
						With an Er:YAG laser under an air water spray at 200 mJ, 20 Hz, tip diameter of 800 nm, a working distance of 0.5 mm	

For the meta-analysis, we only had immediate bond strength (< 48 h) as the outputs. To meet clinical needs, the temporary period time of less than 15-day groups was assessed, which meant 4-month groups were excluded [26]. We analyzed mechanical removal ways, ignoring different parameters applied. We excluded articles of high risk [25, 31]. The sample size input was the number of teeth.

**Fig. 2:**
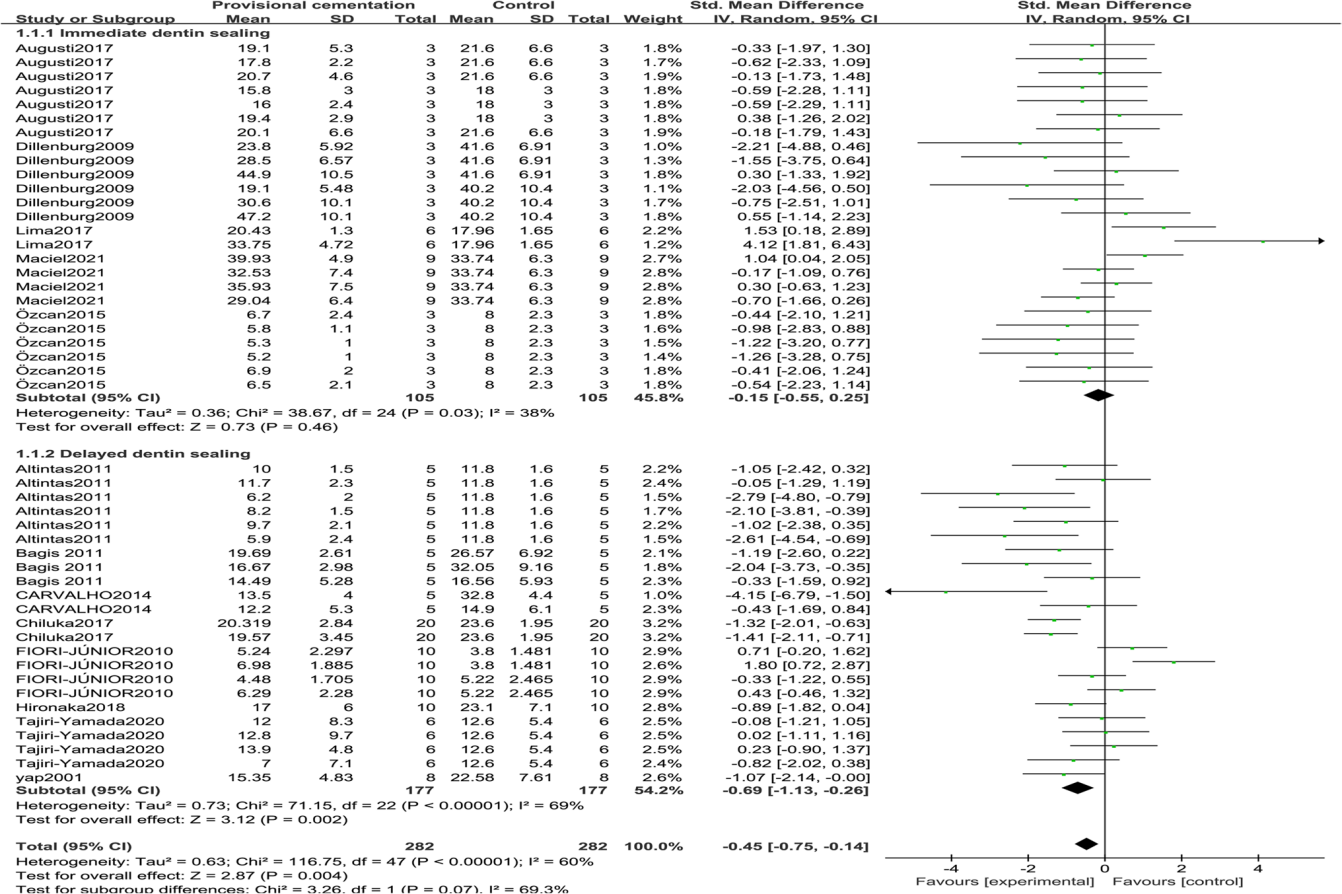
Forest plot of global and subgroups (immediate or delayed dentin sealing) meta-analyses. The immediate (< 48 h) dentin bond strength with and without temporary cementation (experiment and control groups)

**Fig. 3:**
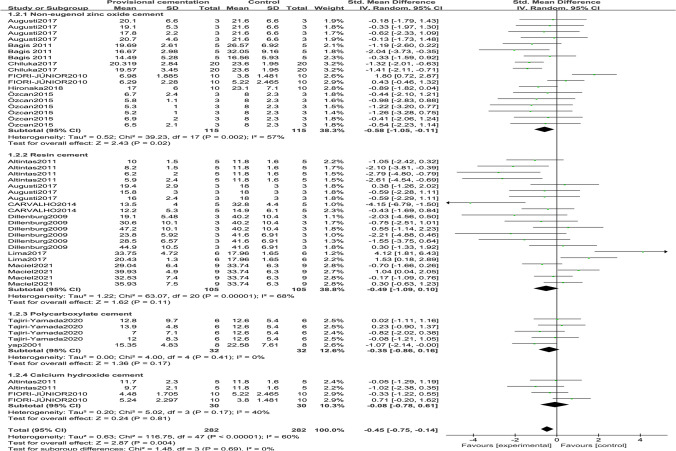
Forest plot of global and subgroups among four temporary cements. The immediate (< 48 h) dentin bond strength with and without temporary cementation (experiment and control groups)

**Fig. 4:**
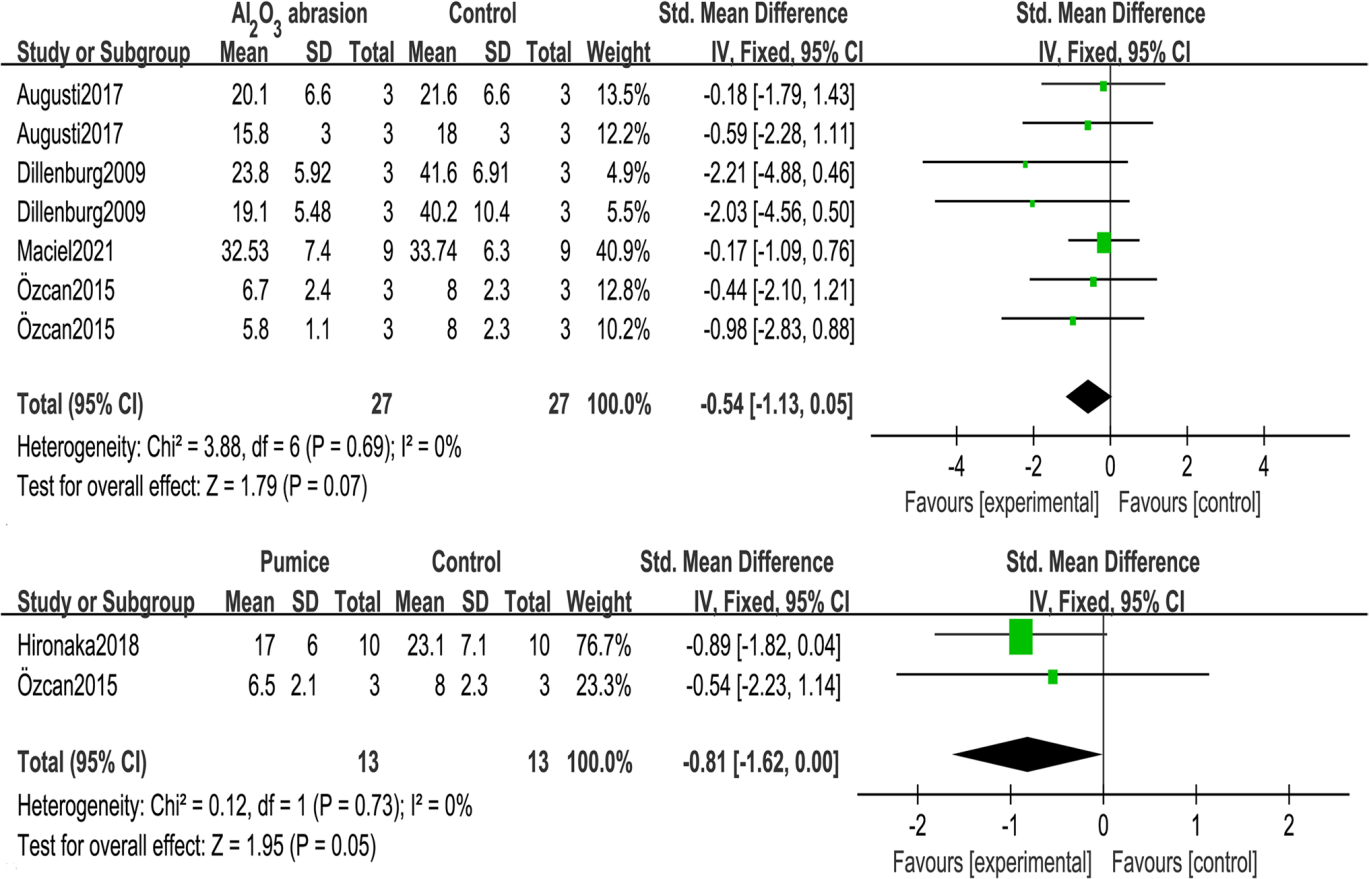
Forest plots of subgroups of two mechanical removal ways (Al_2_O_3_ abrasion, pumice) on immediate (< 48 h) dentin bond strength with and without temporary cementation (experiment and control groups)

**Fig. 5:**
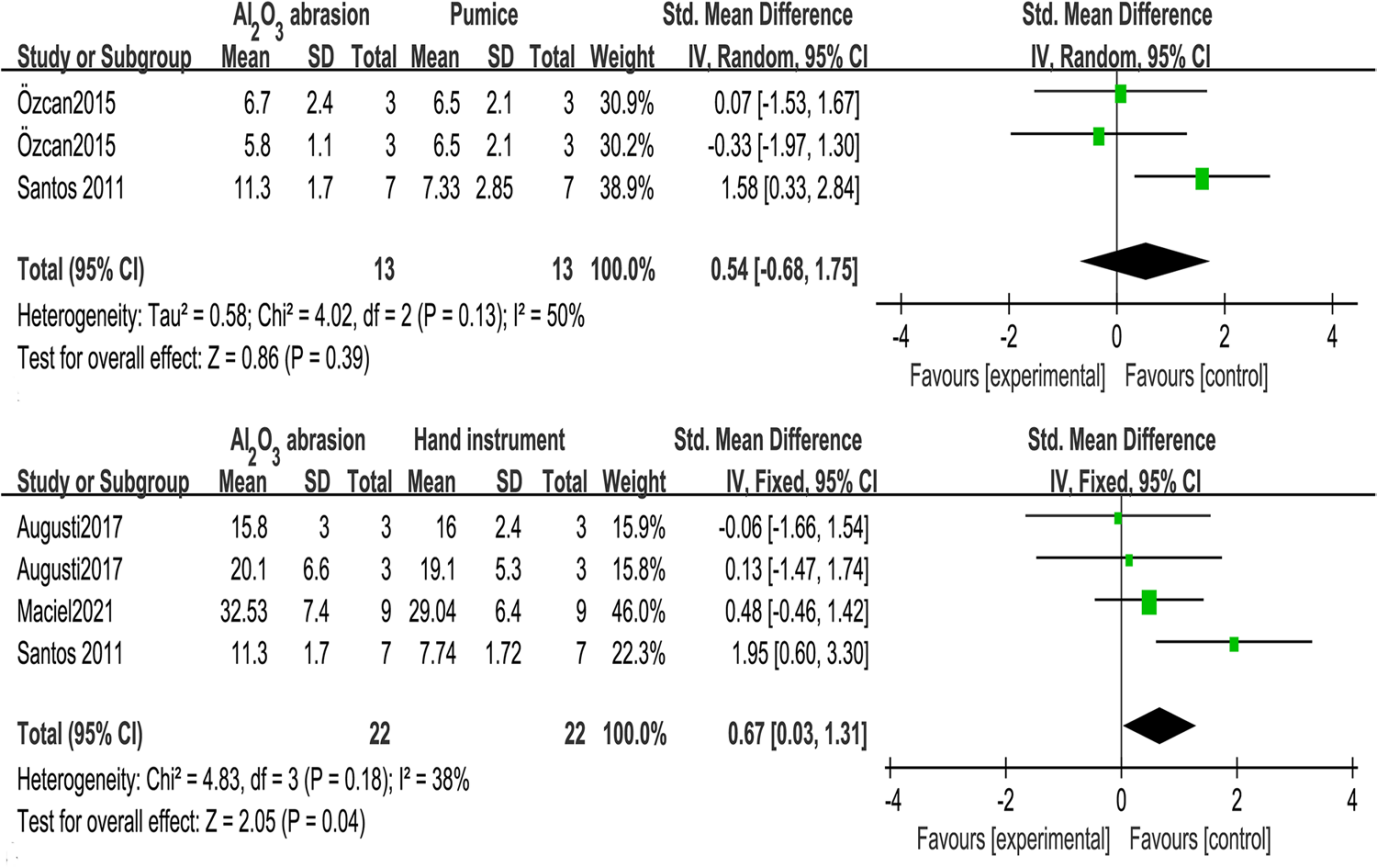
Forest plots of subgroups of mechanical removal way comparisons (Al_2_O_3_ abrasion vs. pumice, Al_2_O_3_ abrasion vs. hand instruments) on immediate (< 48 h) dentin bond strength with temporary cementation

Data from 14 articles underwent meta-analysis. The results of the meta-analysis are shown in Figs. 2, 3, 4, and 5. In Fig. 2, temporary cementation negatively affected the immediate bond strength (*Z*-test *p* = 0.004) by − 0.45 MPa (95% CI =  − 0.75 to − 0.14). However, the negative effect could be mitigated by the IDS strategy so that the temporary cementation had no significant impact on bond strength (*Z*-test *p* = 0.46, 95% CI =  − 0.55 to 0.25). In contrast, under DDS, temporary cementation statistically decreased bond strength (*Z*-test *p* = 0.002) by − 0.69 MPa (95% CI =  − 1.13 to − 0.26). The heterogeneity of IDS was acceptable (*I*^2^ = 38%), while DDS was moderate (*I*^2^ = 69%).

In Fig. 3, four temporary cements were considered, non-eugenol zinc oxide cement, resin cement, polycarboxylate cement, and calcium hydroxide cement. The last three groups indicated no statistically significant impact on immediate bond strength (*Z*-test *p* > 0.05), while non-eugenol zinc oxide cement lowered the bond strength compared with the control group (*Z*-test *p* = 0.02) by − 0.58 MPa (95% CI =  − 1.05 to − 0.11). The first two groups’ intragroup heterogeneity was higher, whereas that of polycarboxylate (*I*^2^ = 0) and calcium hydroxide cements (*I*^2^ = 40%) was lower.

In Fig. 4, the Al_2_O_3_ abrasion and pumice were compared with the control group. The pumice strategy involved was a mixture of flour pumice and water (pumice slurry). Both comparisons were homogeneous (*I*^2^ = 0%). The Al_2_O_3_ abrasion restored the bond strength that decreased after temporary cement contamination (*Z*-test *p* = 0.07), while the bond strength of pumice removal slightly decreased in contrast with the control group (95% CI =  − 1.62 to 0).

In Fig. 5, the hand instruments included periodontal curette [21], hand scaler [23], or excavator [13], which were applied until the dentin surfaces were visually clean. Compared with hand instruments, Al_2_O_3_ abrasion significantly enhanced immediate bond strength (*Z*-test *p* = 0.04) by 0.67 MPa (95% CI = 0.03 to 1.31). However, Al_2_O_3_ abrasion was not superior to pumice on cleaning cements (*Z*-test *p* = 0.39). Their heterogeneity was acceptable (*I*^2^ ≤ 50%).

We removed each article’s findings to assess the sensitivity. In the DDS subgroup, after removing Fiori-Júnior [22], the overall *I*^2^ decreased to 51%. In the resin temporary cement subgroup, after omitting Lima [16], there was a decline in intragroup heterogeneity (*I*^2^ from 68 to 56%) and swift of effect (*Z*-test *p* from 0.11 to 0.007), leading to an 8% drop of overall *I*^2^. The altered result was that resin temporary cement lowered the bond strength by − 0.73 MPa (95% CI =  − 1.26 to − 0.2). The overall effect and heterogeneity were stable by removing others.

## Discussion

The main objective of this review was to assess the influence of IDS or DDS, temporary cement types, and cleaning methods on immediate bond strength. For a conventional indirect restoration, the temporary cement inevitably contaminated collapsed dentin collagen [32], making it difficult to completely remove, especially when it penetrated deeply [30], complying with this finding that the bond strength under DDS significantly declined after temporary cementation. In sensitivity analysis, the study by Fiori-Júnior greatly increased heterogeneity because of its anomalous conclusion that the combination of zinc oxide cement and etch-and-rinse adhesive obtained higher bond strength than the non-contaminated group [22].

On the contrary, IDS eliminated the negative effect of temporary bonding with low heterogeneity, regardless of distinct luting systems and removal ways, which was supported by Augusti [23] and Mine [33]. The success of IDS was that it pre-cured dentin adhesive immediately after tooth preparation and formed a hybrid layer better without contamination from temporary cements or blood [34–36], which was verified by its thick and continuous interfacial zone [12]. By micro-Raman spectroscopy, the particular interface peak (1330 cm^−1^) of IDS revealed a chemical interaction of resin cement and dentin [12]. What is more, the polymerized IDS layer prevented the hybrid layer from degrading and kept it stable over time [26, 32, 37]. The IDS layer, in addition to acting as a stress breaker for external forces [11, 38], also released the stress of polymerization shrinkage, leading to higher fracture resistance and greater survival of veneer [39, 40].

This analysis only targeted immediate bond strength, but some other experiments with various aging processes have also validated the critical role of IDS [11, 25, 32]. Through the Weibull values, the failure predictability and bond durability of IDS outperformed DDS after aging [11]. By simulating over 14-month cyclic loading, though the IDS restored the bond strength after temporary cementation, its Weibull values decreased, suggesting contamination of the first pre-cured IDS layer might have a long-term negative impact on the bond strength [11, 17]. A thicker IDS layer was recommended, considering the effect that Al_2_O_3_ abrasion might weaken the surface of IDS layer [26, 41]. In conclusion, the IDS technique could reduce the negative effects of temporary bonding in the short or long term compared with DDS.

Since temporary cement residue could impede the wetting and infiltrating ability of luting cements [30], cleaning them was required before proceeding to the next step [11]. This analysis concluded that resin-based, polycarboxylate, and calcium hydroxide cements had no significant effect on the immediate bond strength, except for non-eugenol zinc oxide cements that had an adverse impact. The subgroup difference showed no heterogeneity (*I*^2^ = 0), supporting the pooled results, whereas the heterogeneity of polycarboxylate and calcium hydroxide cements subgroups was acceptable (*I*^2^ < 50), but only 2 articles were included. The polycarboxylate cement was chemically bonded to dentin via an ion-exchange mechanism, making it difficult to remove. To adequately remove it, the applications of phosphoric acid (PA) plus NaClO or a cleaner containing 10-methacryloyloxydecyl dihydrogen phosphate (MDP) were more suitable and effective [24].

The intragroup heterogeneity of non-eugenol zinc oxide cement was higher, owing to IDS or DDS selection and inconsistent final luting systems. The acidic primer of self-etching adhesive exacerbated the adverse impact of zinc oxide cement [30], because they might react with each other, impeding resin penetration [25]. The finding was in accordance with another study that the negative effect of self-etching system was stronger than etch-and-rinse procedure after temporary cementation [20]. Conversely, self-adhesive cement attained comparable bond strength before and after zinc oxide cement [30]. After all, when choosing zinc oxide cement, be aware that its performance with self-etching cement was undesirable.

Previous articles have advised against using resin-based temporary cement because of its high risk of bonding sealed dentin [5, 15], making it difficult to remove even by sandblasting [23]. Resin temporary cement would plug the dentinal tubules, interfering with subsequent adhesive penetration [3, 25]. However, this article concluded that the resin-based temporary cement had no significant effect on bond strength with moderate intragroup heterogeneity, most likely due to the exceptional research by Lima [16]. Abnormally, Lima indicated that the resin cement acquired significantly higher bond strength than the control group, possibly because the acrylate-based temporary cement interacted with unreacted monomers in oxygen-inhibited layer and promoted adhesion. After omitting this research, we concluded the opposite result that resin temporary cement was harmful to bond strength under most conditions. When followed by an etch-and-rinse system, resin temporary cement did not significantly undermine the immediate bond strength [14, 21, 23]. But we should avoid it due to its negative effects in most situations.

To enhance bond performance between the contaminated dentin and luting cement, we required to clean effectively [30], which primarily served two purposes: the adequate cleaning of residual cement and the roughening of dentin surface [23], thus promoting the wettability of adhesive. Merely manual instruments (hand scaler, periodontal curette, and excavator) were inefficient procedures to microscopically remove cements [23, 30, 42, 43], especially for resin-based cement [3, 23], so they were often the first step to remove cement, combining with other mechanical or chemical removal ways to prevent the reduction of bond strength [12, 26].

Airborne particle abrasion of Al_2_O_3_ or glycine [13, 21, 23, 42] and Al_2_O_3_ abrasion plus PA produced the highest bond strength values [26]. The present analysis showed that Al_2_O_3_ abrasion outperformed hand instruments on bond strength and achieved the comparable immediate bond strength to the control group. Januario also revealed that Al_2_O_3_ abrasion performed best after a 90-day period of water storage [42]. The probable reason for its advantage was that it created an irregular and rough dentin surface without residual cement, which improved wettability [13, 21, 42], similar to the mechanism of glycine powder [42]. Besides, since silicoated Al_2_O_3_ modified the surface by depositing silica particles, resulting in chemical interaction between silane and resin luting cement, it was applied with silane coupling agent, but which failed to have an advantage over pumice alone [32, 37]. For particle mentioned above, there was no analysis of which particle performed best due to a lack of comparisons. Conversely, abrasions of NaHCO_3_ or CaCO_3_ particles were ineffective in enhancing bond strength [42, 44]. Because NaHCO_3_ abrasion left smear layer and its residue increased superficial pH, the reaction between PA and acidic monomer was interfered [42, 45, 46].

Another popular cleaning method was to apply pumice slurry or fluoride-free pumice paste with a rotary instrument to remove plaque and surface debris, particularly for unfilled adhesives [16, 42, 44]. In this meta-analysis, cleaning with pumice failed to achieve bond strength comparable to the control group, but there was no discernible difference between Al_2_O_3_ abrasion and pumice. Despite this, the application of pumice was discouraged owing to its less reliability than Al_2_O_3_ abrasion [14]. The possible reason was that partial dentin tubules were occluded by particle remnants by the force of rotation [13, 14], leading to less wettability and roughness [42, 44].

As to chemical removal ways, the additional use of PA might lower the bond strength [31], which could be improved by adding NaClO with its deproteination function, dissolving the exposed collagen fibers and allowing the resin to penetrate further [47]. Others also found that the combination or a new cleaner containing MDP did not differ significantly from the control even after 6-month water storage. Not only did hydrophilic and hydrophobic groups of MDP act as a surfactant to clean, but also its remaining phosphoric group could interact with apatite and copolymerize with resin monomers [24]. The combination of PA and NaClO and a cleaner containing MDP were worth developing in terms of removal effectiveness and bond durability.

The limitation of the study was that we only analyzed immediate bond strength (< 48 h) because of the lacking and heterogeneous aging procedures. Second, we compared mechanically cleaning ways based on various parameters that might affect bond strength [48, 49]. Third, the number of similar literature included (only two) was insufficient for four comparisons. In future studies, aging processes and pulpal pressure need to be considered to simulate the oral environment [50, 51]. Further researches are required to determine which specific parameters of removal ways have optimal cleaning effects. Additionally, CAD/CAM technique was prospective for development, making it possible to eradicate negative effects of temporary cementation by fabricating restorations on the same day. Consequently, the null hypothesis in this research was rejected.

## Conclusions

Within the limitations of this analysis, the following conclusions were drawn. (1) IDS was extremely effective in eliminating the negative effects of temporary bonding in the short or long term, regardless of the luting systems and removal methods. (2) Compared with resin-based and non-eugenol zinc oxide cements, polycarboxylate and calcium hydroxide temporary cements led to higher bond strength. Self-etching adhesive would exacerbate the adverse impact of temporary cement. (3) Pumice and hand instrument removal ways failed to clean effectively and reliably, whereas Al_2_O_3_ abrasion achieved the comparable bond strength with the control group and outperformed hand instruments.

## Supplementary Information

Below is the link to the electronic supplementary material.

Supplementary file1 (DOCX 17 KB)
