# Supplementary material for: Effect of temporary cements and their removal methods on the bond strength of indirect restoration: a systematic review and meta-analysis
Source: Clin Oral Investig. 2022 Nov 24;27(1):15–30. doi: 10.1007/s00784-022-04790-6 (PMC9877054; doi:10.1007/s00784-022-04790-6)
Supplement: Supplementary file 1 — Supplementary file1 (DOCX 17 KB) [file 784_2022_4790_MOESM1_ESM.docx]

**Web of science:TS=(Bonding OR Bond OR Bonding efficacy OR Dental bonding OR bond strength OR bonding effectiveness OR Bonding performance OR Bond performance OR adhesive properties OR Micro-tensile strength OR microtensile strength OR Microtensile bond strength OR bonding properties OR microshear bond strength OR shear bond strength OR performance) AND TS=(Dentin*) AND TS=(provisional cement* or temporary cement* or interim cement* or temporary restoration* or provisional restoration* or interim restoration*)**

Pubmed: **(((dentin*[Title/Abstract]) OR (dentin[MeSH Terms])) AND (Bonding[Title/Abstract] OR Bond[Title/Abstract] OR Bonding efficacy[Title/Abstract] OR Dental bonding[Title/Abstract] OR bond strength[Title/Abstract] OR bonding effectiveness[Title/Abstract] OR Bonding performance[Title/Abstract] OR Bond performance[Title/Abstract] OR adhesive properties[Title/Abstract] OR Micro-tensile strength[Title/Abstract] OR microtensile strength[Title/Abstract] OR Microtensile bond strength[Title/Abstract] OR bonding properties[Title/Abstract] OR microshear bond strength[Title/Abstract] OR shear bond strength[Title/Abstract])) AND (provisional cement*[Title/Abstract] OR temporary cement*[Title/Abstract] OR interim cement*[Title/Abstract] OR temporary restoration*[Title/Abstract] OR provisional restoration*[Title/Abstract] OR interim restoration*[Title/Abstract])**

Cochrane

#1 (Dentin*):ti,ab,kw (Word variations have been searched) 4872

#2 (Bonding OR Bond OR Bonding efficacy OR Dental bonding OR bond strength OR bonding effectiveness OR Bonding performance OR Bond performance OR adhesive properties OR Micro-tensile strength OR microtensile strength OR Microtensile bond strength OR bonding properties OR microshear bond strength OR shear bond strength):ti,ab,kw (Word variations have been searched) 5871

#3 (provisional cement* or temporary cement* or interim cement* or temporary restoration* or provisional restoration* or interim restoration*):ti,ab,kw (Word variations have been searched) 580

#4 #1 and #2 and #3 22

Embase

| #4 | #1 AND #2 AND #3 | **85** |
| --- | --- | --- |
| #3 | dentin*:ab,ti | **34003** |
| #2 | 'provisional cement*' OR 'temporary cement*' OR 'interim cement*' OR 'temporary restoration*' OR 'provisional restoration*' OR 'interim restoration*':ab,ti | **1651** |
| #1 | 'bonding'/exp OR bonding OR 'bond'/exp OR bond OR 'bonding efficacy' OR 'dental bonding'/exp OR 'dental bonding' OR 'bond strength'/exp OR 'bond strength' OR 'bonding effectiveness' OR 'bonding performance' OR 'bond performance' OR 'adhesive properties' OR 'micro-tensile strength' OR 'microtensile strength' OR 'microtensile bond strength'/exp OR 'microtensile bond strength' OR 'bonding properties' OR 'microshear bond strength' OR 'shear bond strength':ab,ti | **404286** |

National Repository of Grey Literature, NRGL keyword: dentin bonding 2
